# Alteration of the IL-33-sST2 pathway in hypertensive patients and a mouse model

**DOI:** 10.1038/s41440-019-0291-x

**Published:** 2019-06-24

**Authors:** Xiaoyun Yin, Huajun Cao, Yingjie Wei, Hui-Hua Li

**Affiliations:** 1 State Key Laboratory of Cardiovascular Disease, Fuwai Hospital, National Center for Cardiovascular Diseases, Chinese Academy of Medical Sciences and Peking Union Medical College, Beijing, 100037 People’s Republic of China; 2 Department of Cardiology, Institute of Cardiovascular Diseases, First Affiliated Hospital of Dalian Medical University, Dalian, 116011 People’s Republic of China; 3 School of Public Health, Dalian Medical University, Dalian, 116044 People’s Republic of China

**Keywords:** Hypertension, IL-33, sST2, ST2L, Peripheral blood mononuclear cells

## Abstract

Inflammatory cells play an important role in the occurrence of hypertension. Recent studies have demonstrated that interleukin-33/suppression of tumorigenicity 2 (IL-33/ST2) signaling plays a critical role in the pathogenesis of several cardiovascular diseases. We aimed to evaluate the association of IL-33 and its receptor levels with the occurrence of hypertension in angiotensin II (Ang II)-infused mice using microarray analysis and validated our results in human specimens. Male wild-type mice were infused with Ang II (1500 ng/kg/min) for 1, 3 and 7 days. Patients with essential hypertension (EH) (*n* = 166) and healthy control subjects (*n* = 306) were enrolled. Levels of IL-33 and ST2 mRNAs in serum and peripheral blood mononuclear cells (PBMCs) were analyzed by Luminex assay or ELISA and qPCR analysis. We found that IL-33 expression was significantly increased in the aortas of mice receiving Ang II infusion compared with that of control mice. In contrast, the levels of IL-33 in serum and PBMCs were not significantly different between hypertensive patients and normal controls. However, the levels of soluble ST2 (sST2) in serum and PBMCs were markedly higher in hypertensive patients than in controls (*P* < 0.001 and *P* = 0.014, respectively). In addition, the ST2L level in PBMCs was also significantly decreased in hypertensive patients (*P* = 0.028). Further, logistic analysis showed that the odds ratios of having hypertension based on sST2 levels in serum and PBMCs were 9.714 and 2.244 (*P* = 0.013 and *P* = 0.024, respectively) compared with the control group. Above all, sST2 acted as a risk factor for the occurrence of hypertension and may be a promising novel predictive marker for EH.

## Introduction

Hypertension is a significant public health problem worldwide and affects more than 30% of the population and 70% of elderly adults. Epidemiological studies indicate that hypertension is one of the most important risk factors for cardiovascular diseases, including atherosclerosis, stroke, and coronary heart disease [1–3]. Until now, the etiology of most cases of adult hypertension remains unclear. However, abnormalities in the central nervous system, vasculature and kidney have been implicated in the pathogenesis of hypertension [4]. Accumulating data from animals and human patients demonstrate that hypertension is a low-grade inflammatory disease. Immune cells can infiltrate multiple organs, thereby causing dysfunction and leading to elevation of blood pressure. Indeed, monocyte/macrophages and lymphocytes are increased in the kidney and vasculature of animal models of experimental hypertension and in humans with hypertension [5, 6], suggesting that the inflammatory effect of PBMCs plays a critical role in vascular dysfunction and hypertension [1–3]. Thus, the identification of key inflammatory mediators may provide new insights into the pathogenesis of EH [7].

Interleukin IL-33, a newly identified member of the IL-1 cytokine family, is a nuclear-associated multifunctional cytokine that acts as an “alarmin” [7, 8]. ST2 is the receptor for IL-33 and has two forms, the soluble (sST2, also known as IL1RL1-a) and transmembrane (ST2L, also known as IL1RL1-b) forms [9–11]. IL-33 is mainly released by damaged, necrotic barrier cells or stimulated leukocytes [8] and participates in a wide range of immune diseases, including chronic inflammation and connective tissue diseases, as well as allergies [12]. Several studies have reported that IL-33, sST2 and ST2L are also expressed in human coronary and carotid plaques [13, 14]. IL-33 acts through ST2 signaling by binding to ST2L. The cardioprotective effect of IL-33 is abolished in ST2-null mice [15]. IL-33 also reduces the formation of foam cells and the development of atherosclerosis by blunting the Th1 and M1 immune responses, while sST2 has deleterious effects by sequestering IL-33 [16]. Moreover, IL-33 prevents cardiomyocyte apoptosis, decreases myocardial fibrosis and myocyte hypertrophy and impedes the atherosclerotic process [12, 17–19]. IL-33 also reduces heart infarct size and improves left ventricular function [15]. Thus, IL-33 plays critical roles in cardiovascular diseases.

IL-33 is directly regulated by mechanical stretching of the myocardium and has a protective effect on the heart [20]. An elevated IL-33 level in patients with pulmonary arterial hypertension has a protective effect by promoting Th1 to Th2 T-cell cytokine skewing [20]. Further, hypertension-increased myocardial pressure also leads to elevated circulating IL-33. sST2 decreases the tissue availability of IL-33 and thus blocks the anticardiac hypertrophy and protective effects against atherosclerosis by binding to it [19]. In patients with chronic and acute decompensated heart failure, serum sST2 levels are increased and strongly associated with both disease severity and mortality [21, 22]. However, no significant correlations have been reported among IL-33, sST2 and primary hypertension. We aimed first to detect the expression of IL-33 in the aortas of mice with hypertension. We further verified the expression of IL-33 and its receptors in serum and PBMCs from patients with EH and investigated their possible relationships with EH.

## Methods

### Animal experiments

Wild-type male mice aged 8 to 10 weeks (C57BL/6J) were purchased from Jackson Laboratory (Sacramento, CA, USA). The mice were divided into four groups. According to previous studies [23], we set the number of mice in each group at *n* = 3. The control group was injected with normal saline by osmotic mini-pumps (Alzet Model 1007D, Cupertino, CA, USA) at a dose of 1500 ng/kg/min. Experimental groups 1 to 3 were hypertension model groups induced by subcutaneous infusion of Ang II (Sigma-Aldrich, St. Louis, MO, USA) at a dose of 1500 ng/kg/min for 1, 3 or 7 days, respectively.

The aortic tissues were collected for microarray analysis and quantitative real-time polymerase chain reaction (qPCR) analysis. The following primers were used: mouse IL-33 forward, 5′-ATTTCCCCGGCAAAGTTCAG-3′ and reverse, 5′-AACGGAGTCTCATGCAGTAGA-3′; and mouse GAPDH forward, 5′-AGGTCGGTGTGAACGGATTTG-3′ and reverse, 5′-GGGGTCGTTGATGGCAACA-3′. These primers were synthesized by Sangon Biotech (Shanghai, CHN). The specificity of the amplification was determined by melting curve analysis. The relative fold change in each gene was calculated using the 2(−ΔΔC(T)) method [24]. The experimental protocol was approved by the Animal Care and Use Committee of Dalian Medical University and conformed to the US National Institutes of Health Guide for the Care and Use of Laboratory Animals.

### Human samples collection

Since February 2017, we have recruited patients with essential hypertension from the Division of Cardiology of the First Affiliated Hospital of Dalian Medical University in Liaoning Province. Patient eligibility included two parts: 1) newly diagnosed hypertension, or 2) the patient was previously diagnosed with essential hypertension but did not follow the doctor’s instructions for medication. Essential hypertensive patients should be diagnosed according to the guidelines for the diagnosis and treatment of ESC hypertension in 2017 [25]. All patients with secondary hypertension, coronary artery disease, heart failure, stroke, valvular heart disease, collagen disease, diabetes mellitus, advanced liver disease, renal failure, malignant disease, sepsis or other inflammatory diseases, recent trauma history, mental disorders and autoimmune diseases were excluded.

During the same period, age-matched healthy controls were recruited at the Health Examination Center of the same hospital. Systolic blood pressure should be below 120 mmHg, and diastolic blood pressure should be below 80 mmHg. Exclusion criteria were as follows: 1) There was hypertension among immediate relatives. 2) Patients with hypertension, coronary artery disease, heart failure, stroke, valvular heart disease, collagen disease, diabetes, advanced liver disease, renal failure, malignant diseases, sepsis or other inflammatory diseases.

All participants were required to complete a systematic collection of medical history, including smoking history, drinking history, and medication history, and to complete basic clinical hematology examinations, including routine blood, routine urine, liver function, kidney function, blood glucose, and uric acid. Chest and abdominal imaging examinations were also required. By inquiring about the medical history, we tried to select the subjects who had not used any drugs within 6 months before the start of this study in both groups. People with a recent history of trauma and people with psychiatric and autoimmune diseases were not included.

We conducted pre-experiments and used a two-sample *t*-test method to estimate the sample size using PASS software (version 16, PASS, Utah, USA). By October 2018, 166 patients with essential hypertension and 306 healthy controls were included in the case-control study. The research was carried out according to the principles of the Declaration of Helsinki. This study was approved by the Human Ethics Committee of the First Affiliated Hospital of Dalian Medical University. Written informed consent was obtained from each patient.

Fasting blood samples were obtained with a 21-gauge needle for a clean venipuncture of an antecubital vein while participants were in a recumbent position as described previously [26]. Five milliliters of peripheral venous blood was collected in a vacuum tube without any anticoagulant for serum separation. Another 5 ml of peripheral venous blood was collected into an EDTA anticoagulant tube. Peripheral blood mononuclear cells (PBMCs) were prepared by Ficoll density gradient centrifugation for analysis. Serum and PBMCs were kept at −80 °C until further use.

### Magnetic Luminex assay and enzyme-linked immunosorbent assay (ELISA)

Human serum IL-33 was measured using a Human Premixed Multi-Analyte Kit (L121325, R&D Systems, Minneapolis, MN, USA) according to the manufacturer’s instructions. Levels of human serum ST2 were examined using a human ST2 ELISA kit (DST200, R&D Systems, Minneapolis, MN, USA). All reagents were prepared following the manufacturer’s protocol.

### qPCR analysis

Total RNA was extracted using an RNA extraction kit (R6814-01, Omega, Norcross, GA, USA) following the manufacturer’s instructions. Genomic DNA was digested, and cDNA was synthesized with a PrimeScript™ RT reagent Kit with gDNA Eraser (RR047Q, Takara, Tokyo, JPN). qPCR was conducted with TB Green Premix Ex Taq II (RR820A, Takara, Tokyo, JPN) in a 7500-Fast Real-Time PCR System. The following primers were used: human IL-33 forward, 5′-TGACGGTGTTGATGGTAAGAT-3′ and reverse, 5′-AGCTCCACAGAGTGTTCCTTG-3′; human sST2 forward, 5′-GGCACACCGTAAGACTAAGTAG-3′ and reverse, 5′-CAATTTAAGCAGCAGAGAAGCTCC-3′; human ST2L forward, 5′-ATGTTCTGGATTGAGGCCAC-3′ and reverse, 5′-GACTACATCTTCTCCAGGTAGCAT-3′; and human GAPDH forward, 5′-AGCGAGATCCCTCCAAAATC-3′ and reverse, 5′-GGCAGAGATGATGACCCTTT-3′. These primers were synthesized by Sangon Biotech (Shanghai, CHN). The specificity of the amplification was determined by melting curve analysis. The relative fold change in each gene was calculated using the 2(−ΔΔC(T)) method [24].

## Statistical analysis

The Kolmogorov–Smirnov test was used to determine the distribution of the data. Normally distributed data were expressed as the mean ± SD and non-normally distributed data as the median and interquartile range (IQR): 25th to 75th percentiles. PASS (version 16, PASS, Utah, USA), SPSS (version 24.0, SPSS, Chicago, IL, USA) and GraphPad Prism Program (version 6.0, GraphPad, San Diego, CA, USA) were used to perform statistical analysis. Student’s *t* test was used when comparing two groups of normally distributed data. The continuous variables of characteristics were log-transformed to establish normality of distribution when necessary. When the data were still non-normally distributed after logarithmic transformation, the Mann–Whitney *U* test was used. Pearson’s correlation was used to calculate the correlations between serum sST2 and several parameters, including serum IL-33, SBP and DBP. Binary logistic regression was used to analyze the risk factors of essential hypertension. Candidate covariates included age, sex, BMI, triglycerides, fasting blood glucose and white blood cell count. *P* < 0.05 indicated statistical significance.

## Results

### IL-33 expression is upregulated in aortas of Ang II-infused mice

**Fig. 1:**
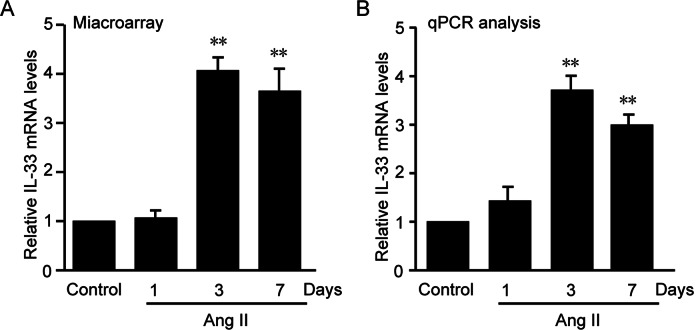
Increased levels of IL-33 mRNA in aortic tissues in an Ang II-induced hypertension model by subcutaneous infusion of Ang II as shown by microarray analysis and qPCR analysis. **a** Relative IL-33 mRNA levels in microarray analysis. The abscissa coordinates were the control group, Ang II infusion for 1 day, Ang II infusion for 3 days and Ang II infusion for 7 days. The ordinate was the relative expression levels of IL-33 mRNAs in the microarray analysis. **b** Relative IL-33 mRNA levels in qPCR analysis. The abscissa coordinates were the control group, Ang II infusion for 1 day, Ang II infusion for 3 days and Ang II infusion for 7 days. The ordinate was the relative expression levels of IL-33 mRNAs in qPCR analysis. **P < 0.01 compared to control. qPCR quantitative real-time polymerase chain reaction

To investigate the relationship between IL-33 and hypertension in mice, we first infused male wild-type mice with Ang II (1500 ng/kg/min) for 1–7 days to induce hypertension and then performed microarray analysis. We found that IL-33 expression was markedly upregulated in Ang II-infused aortas at different time points compared with controls (Fig. 1a). Increased expression of IL-33 was further confirmed by quantitative PCR analysis (Fig. 1b).

### Clinical and biochemical characteristics of the study population

A total of 472 participants were enrolled, including 166 hypertension patients and 306 healthy controls. The clinical and biochemical characteristics of the two groups are shown in Table 1. There was no significant difference in age, heart rate, total cholesterol, LDL, or HDL between the two groups. However, hypertensive patients had higher triglycerides (1.81 ± 1.34 vs 1.32 ± 0.81; *P* < 0.001), fasting blood glucose (5.11 ± 0.47 vs 4.85 ± 0.41; *P* *<* 0.001), white blood cell count (5.95 ± 1.32 vs 5.60 ± 1.13; *P* *=* 0.004), and BMI (24.88 ± 2.85 vs 24.21 ± 2.76; *P* *=* 0.013) compared to the control group. The percentages of each sex in the two groups was significantly different (male: 64.46% vs 42.48%; *P* *<* 0.001).

**Table 1:** Clinical and biochemical characteristics of control subjects and hypertensive patients

Characteristics	Normotensive Controls (*n* = 306)	Hypertensive Patients (*n* = 166)	*P* Value
Age (yrs)	55.11 ± 7.93	54.29 ± 9.31	0.334
Male (*n*, %)	130 (42.48)	107 (64.46)	<0.001
Systolic blood Pressure (mm Hg)	111.61 ± 7.68	153.13 ± 13.38	<0.001
Diastolic blood Pressure (mm Hg)	70.15 ± 6.70	92.61 ± 10.32	<0.001
Heart rate, bpm	68.31 ± 7.01	69.33 ± 6.91	0.129
Total Cholesterol (mmol/L)	5.31 ± 0.92	45.46 ± 0.99	0.097
LDL (mmol/L)	2.86 ± 0.66	2.98 ± 0.66	0.085
HDL (mmol/L)	1.58 ± 0.34	1.54 ± 0.38	0.199
Triglycerides (mmol/L)	1.32 ± 0.81	1.81 ± 1.34	<0.001
Fasting blood glucose (mmol/L)	4.85 ± 0.41	5.11 ± 0.47	<0.001
White blood cell count (10^9/L)	5.60 ± 1.13	5.95 ± 1.32	0.004
BMI	24.21 ± 2.76	24.88 ± 2.85	0.013

All the data are expressed as mean ± SD, except male [*n* (%)]

*HDL* high-density lipoprotein, *LDL* low-density lipoprotein, *BMI* body mass index

### The serum levels of IL-33 and sST2

**Fig. 2:**
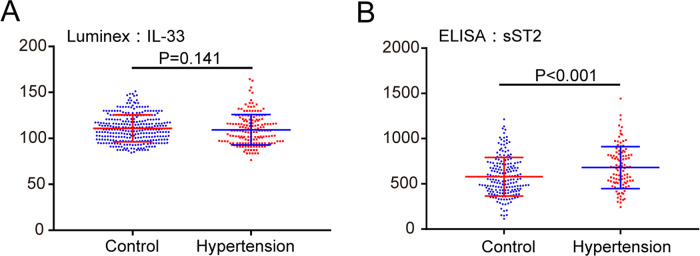
Levels of IL-33 and sST2 in serum in hypertensive patients and control groups. Data are represented as the median (IQR). **a** Luminex assay showed that there was no significant difference in serum IL-33 levels between hypertensive patients and controls (median: 108.34 ng/ml and 109.65 ng/ml, *P* = 0.141). **b** ELISA indicated that sST2 levels were markedly elevated in hypertensive patients compared to normal controls (median: 663.84 vs 549.05 ng/mL, *P* < 0.001). IQR interquartile range

We next examined the levels of IL-33 and sST2 in human peripheral blood serum. Data are represented as the median (interquartile range (IQR)). Luminex assay showed that there was no significant difference in serum IL-33 levels between the hypertension group and the control group (hypertension: median 108.34 ng/ml, IQR: 94.8–119.84; control: median 109.65 ng/ml, IQR: 98.82–120.69; Fig. 2a). In contrast, an ELISA indicated that sST2 levels were markedly elevated at 663.84 ng/ml (median, IQR: 514.29–818.97) in hypertensive patients compared with 549.05 ng/ml (median, IQR: 426.62–720.92) in normal controls (*P* < 0.001, Fig. 2b). The IL-33/sST2 ratio was also significantly decreased in hypertensive patients compared to normal controls (hypertension: median 0.17 ng/ml, IQR: 0.13–0.24; control: median 0.20 ng/ml, IQR: 0.14–0.28; *P* < 0.001).

### The levels of IL-33, sST2 and ST2L in PBMCs

**Fig. 3:**
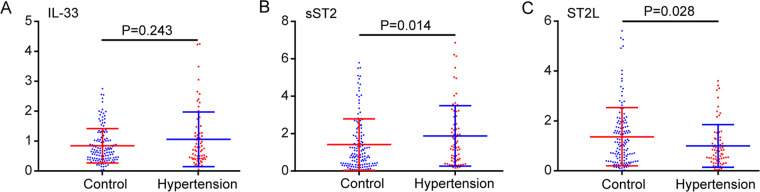
Levels of IL-33, sST2 and ST2L mRNAs in PBMCs in hypertensive patients and control groups. Data are represented as the median (IQR). **a** There was no significant difference in IL-33 mRNA levels between hypertensive patients and normal controls (median: 0.75 vs 0.71; *P* = 0.243). **b** mRNA levels were significantly increased in hypertensive patients compared to normal controls (median: 1.32 vs 0.92; *P* = 0.014). **c** ST2L mRNA levels were markedly decreased in hypertensive patients compared to normal controls (median: 0.78 vs 1.12; *P* = 0.028)

To measure the mRNA levels of IL-33, sST2 and ST2L in PBMCs, we performed qPCR analysis. Data are represented as the median (interquartile range (IQR)). There was no significant difference in IL-33 mRNA levels between hypertensive patients and normal controls (hypertension: median 0.75, IQR: 0.42–1.36; control: median 0.71, IQR: 0.41–1.05; *P* *=* 0.243, Fig. 3a). While sST2 mRNA levels were significantly increased at 1.32 (median, IQR: 0.57–2.80) in hypertensive patients compared to 0.92 (median, IQR: 0.38–1.92) in normal controls (*P* *=* 0.014, Fig. 3b), ST2L mRNA levels were markedly decreased at 0.78 (median, IQR: 0.44–1.28) in hypertensive patients compared with 1.12 (median, IQR: 0.45–1.90) in normal controls (*P* *=* 0.028, Fig. 3c).

### Levels of IL-33, sST2 and ST2L in relation to hypertension

Serum IL-33 was negatively correlated with serum sST2 in Pearson’s correlation (*r* = −0.203, *p* < 0.01). In contrast, serum sST2 was positively correlated with systolic and diastolic blood pressure (*r* = 0.248, *p* < 0.01 and *r* = 0.273, *p* < 0.01, respectively).

### Logical analysis of risk factors for hypertension

We used a binary logistic regression model to assess the risk of IL-33, SST2 and ST2L levels in serum or PBMCs in the occurrence of hypertension. After adjusting for sex, age, BMI, triglyceride, fasting blood glucose and white blood cell count, we found that serum sST2 was a risk factor for EH (OR (odds ratio) = 9.714, *P* = 0.013). Similarly, sST2 mRNA in PBMCs was also a risk factor for EH (OR = 2.244, *P* = 0.024) (Table 2).

**Table 2:** Binary logistic regression model of IL33, sST2 and ST2L levels

Variables	Odds Ratio (95% CI)	*P* Value
Serum IL-33 (ng/ml)	0.174 (0.005–5.781)	0.328
Serum sST2 (ng/ml)	9.714 (1.618–58.321)	0.013
PBMCs IL-33 mRNA	1.293 (0.515–3.245)	0.584
PBMCs sST2 mRNA	2.244 (1.111–4.533)	0.024
PBMCs ST2L mRNA	0.504 (0.250–1.016)	0.055

All variables were adjusted for sex, age, BMI, triglycerides, blood glucose and white blood cell

*IL-33* interleukin-33, *ST2* suppression of tumorigenicity 2, *sST2* soluble ST2, *ST2L* transmembrane ST2, *PBMCs* peripheral blood mononuclear cells

## Discussion

Accumulating evidence has shown that circulating sST2 not only has a relationship with cardiovascular diseases, including acute myocardial infarction (MI) [15], pulmonary hypertension [20], coronary artery disease [27] and heart failure [21, 22] but also plays a role in many other systemic diseases, including asthma [28], obesity [29], type 2 diabetes [30], diabetic kidney disease [31], tumor [32], alcoholic liver disease [33], and Alzheimer’s disease [34]. Most often, sST2 involvement appeared to be better predictive than IL-33 due to its higher levels and stability. However, the association between sST2 and EH remains unclear.

In the present study, using microarray analysis and qPCR analysis, we showed that IL-33 expression was significantly increased in the aortas of mice receiving Ang II infusion for 1–7 days. It has been reported that IL-33 and its receptor ST2 are both expressed in human endothelial cells [35], and IL-33 is also expressed in human atherosclerotic plaques [13]. ST2L is expressed on Th2 and mast cells but not on T1 cells. IL-33 drives the production of Th2 cytokines [36] and causes a shift in the immunological response from Th1 to Th2 [18]. As atherosclerosis is predominantly a Th1-driven process [37], it may imply that IL-33 might have potential vascular effects. We speculated that when hypertension occurs, the vasculature undergoes mechanical stretching, and the expression of IL-33 in the cells increases. While this increase may protect blood vessels, it may contribute to endothelial dysfunction, early atherosclerosis and vascular injury by promoting angiogenesis, vascular permeability, and endothelial activation with the expression of vascular adhesion molecules [13, 38]. In this study, we used Luminex assay and ELISA to detect the level of IL-33 in human serum and PBMCs. Unexpectedly, no significant change in IL-33 was found between hypertensive patients and controls. The inconsistency may be explained by tissue and species differences or the complex mechanisms of homeostatic balance in humans.

In humans, the levels of sST2 but not IL-33 in serum and PBMCs were significantly elevated in hypertensive patients compared with normal controls (Figs. 2 and 3). Serum sST2 was positively correlated with systolic and diastolic blood pressure by Pearson’s correlation analysis. Serum sST2 and sST2 mRNA in PBMCs were both risk factors for EH, as shown by logistic regression analysis (Table 2). The risk of EH was increased 8.714-fold with the increase in serum sST2 per unit, and the risk of EH was increased 1.244-fold with the increase in sST2 mRNA per unit in PBMCs. The serum IL33/sST2 ratio was also significantly decreased in hypertensive patients compared to normal controls, supporting that the protective effects of IL-33 may be overwhelmed by concurrently elevated levels of sST2 when hypertension occurs. sST2 may work as a reservoir for IL-33 and increase the circulation half-life of IL-33 [17]. Previous clinical studies have demonstrated the prognostic role of sST2 in many cardiovascular diseases. The sST2 level increases immediately after infarction and is independently associated with death [39, 40]. sST2 was most elevated in acute aortic dissection patients and showed better diagnostic function over cardiac troponin I or D-dimer [41]. The sST2 level was correlated with disease severity as a significant predictor of clinical worsening in patients with pulmonary arterial hypertension [20, 42]. The mechanism of sST2 increase in heart disease remains unclear. sST2 increases in response to acute and chronic cardiomyocyte strain [17]. Stress caused by high blood pressure stimulates sST2 protein secretion from cardiac, aortic and coronary endothelial cells [14]. A significant correlation between sST2 and blood pressure has not been reported, likely due to the presence of acute heart failure or acute coronary syndrome in patient cohorts. Other mechanisms of the diseases may predominantly determine both BP and sST2 levels. Therefore, we excluded other cardiovascular diseases in the patient group in our cohort study, so the correlation between sST2 and EH was more evident. Therefore, serum sST2 and sST2 mRNA in PBMCs played a predictive role in vessel stress and the occurrence of EH.

The ST2L receptor is the transmembrane form for IL-33. The ST2L mRNA level was markedly decreased, indicating that the transcription of ST2L and expression of ST2L on the cell surface was decreased. ST2L heterodimerizes with IL-1-receptor accessory protein (IL-1RAcP) to form a signaling complex with MyD88, IRAK and TRAF6 in the presence of IL-33 [43–45], leading to NF-kB and AP-1 activation, which then controls the transcriptional activation of genes involved in inflammation, differentiation, proliferation and apoptosis. Moreover, SIGIRR has recently been shown to negatively regulate IL-33/ST2L signaling [46]. Increased sST2 expression can act as a decoy receptor binding to circulating IL-33 in serum and decrease the amount of IL-33, which can bind with ST2L. This finding is consistent with our conclusion that serum IL-33 was negatively correlated with serum sST2. As a result, increased sST2 weakens the role of the IL-33-ST2L signaling pathway and thus reduces the vascular and cardiac protective effect of IL-33, which may lead to the occurrence of EH.

Above all, we found a significant increase in sST2 in serum and PBMCs in patients with hypertension. In contrast, the levels of IL-33 in serum and PBMCs and ST2L in PBMCs did not change significantly after logistic analysis. The IL-33-ST2L pathway in PBMCs may have a protective effect on EH. Given the alteration of the IL-33-sST2 pathway in hypertension, we conclude that sST2 acts as a risk factor for the occurrence of EH and may represent a promising novel marker for EH prediction.
